# Dielectric microspheres enhance microscopy resolution mainly due to increasing the effective numerical aperture

**DOI:** 10.1038/s41377-022-01056-4

**Published:** 2023-01-11

**Authors:** Arash Darafsheh, Vahid Abbasian

**Affiliations:** Department of Radiation Oncology, Washington University School of Medicine in St. Louis, St. Louis, MO 63110 USA

**Keywords:** Interference microscopy, Imaging and sensing

## Abstract

Microsphere-assisted microscopy utilizing a microsphere in immediate proximity of the specimen boosts the imaging resolution mainly as a result of an increase in the effective numerical aperture of the system.

Light microscopes are one of the most widely used tools for sample inspection in life and material sciences. Their achievable spatial resolution, however, is fundamentally limited to ~*λ*/(2 NA) due to the diffraction of light waves, in which *λ* is the wavelength of light and NA=*n* Sin*θ* is the numerical aperture of the system where *θ* is the maximum collection half-angle of light by the lens. Improving microscopy resolution beyond this limit or even enhancing the resolution of a given system to reach this limit without significant modifications would be of significant interest for various applications.

It has been shown in the past decade that micron-scale dielectric spheres and cylinders can be used as supplementary lenses to improve microscopy resolution, a technique termed “microsphere-assisted microscopy” (MAM)^1^. MAM is a simple, yet efficient approach in which a microsphere (MS) is placed in the immediate vicinity of a specimen, as schematically shown in Fig. 1a. Focusing the objective lens on the virtual image formed underneath the specimen by the MS enables microscopy with higher resolution and magnification compared to using the objective lens without the MS (*θ*_2_ > *θ*_1_ in Fig. 1a). MAM can be performed with low-index (*n*~1.5) MSs^2^ as well as high-index (*n* > 1.9) MSs^3^ placed in a background medium with a lower refractive index than that of the microsphere^4^. The latter approach would allow fabrication of novel resolution-improving optical devices^5^. MAM is a versatile technique and can be incorporated with different microscopes, such as fluorescent^6,7^, confocal^8^, and two-photon^9^ setups. MAM can also be extended to interference and digital holographic microscopies for 3D imaging with enhanced resolution. This can be simply achieved in an interferometric arrangement, by placing the MS within the working distance of either a conventional objective lens^10–15^ or a dedicated interferometric objective lens (Fig. 1b)^16,17^.

**Fig. 1:**
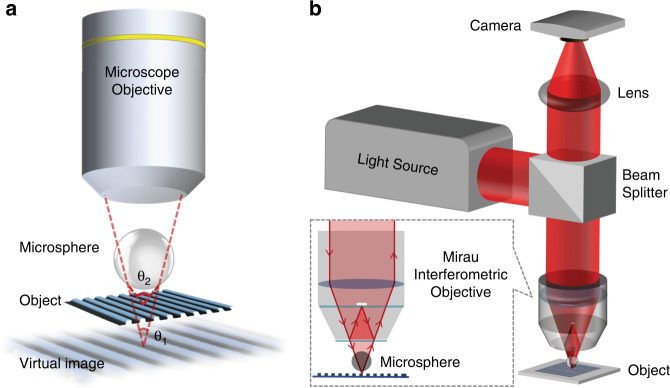
Microsphere-assisted microscopy. Schematic of MAM: (**a**) integration with a conventional microscope objective, and (**b**) extension to 3D microscopy in combination with an interferometric objective lens

MAM has well matched up with interferometric-based approaches, such that both have benefited from each other’s features. Although interferometric arrangements have significantly higher axial resolution compared to conventional microscopy, their lateral resolution would still be restricted by the diffraction limit. However, MAM combined with interferometric setups has shown promising results in producing 3D label-free images with enhanced lateral resolution. Additionally, MAM has shown its potential to be easily integrated with other resolution-enhancing schemes, such as structured and oblique illumination, to achieve even further resolution enhancements in 3D microscopy systems^18,19^. Microsphere-assisted interference microscopy provides an inexpensive and non-destructive imaging technique for 3D surface metrology^17,20^ and microsphere-assisted digital holographic microscopy (DHM) brings forward a cost-effective and easy-to-implement high-quality quantitative phase imaging approach in transmission and reflection modes^21^. The advantages of MAM over other resolution enhancement techniques for DHM are further featured;^22,23^ in MAM, due to the symmetric structure of the MS, the resolution enhancement is achieved simultaneously in all spatial directions, which is extremely important in real-time imaging of specimens with rapid dynamics^12,24^. On the other hand, MAM benefits from DHM’s advantageous features. Adding a spherical lens to a conventional microscope introduces non-negligible spherical aberrations^1^. It also brings about a curved deformation in the measured phase in interference microscopy, which in turn further sacrifices the effective field-of-view (FoV)^13^. However, in DHM, thanks to the phase compensation possibility, the effect of any possible contaminations and aberrations in the optical train on the final data can be removed. To this end, phase information of the reference hologram (taken for no-sample state in the setup) is subtracted from the object phase during the numerical reconstruction process^25^. Another challenging issue in MAM that can be less important in microsphere-assisted DHM is precise positioning of the MS and in some cases preserving the relative sample-to-microsphere distance^26^ during the imaging. However, in DHM, it is consistently possible to be numerically focused on the image plane post-recording of the digital hologram to achieve the best possible imaging quality^27^. Nevertheless, similar to all kinds of MAM, microsphere-assisted interferometric-based approaches also suffer from the limited FoV, which still needs to be addressed in a sustainable manner.

The open questions and challenges in MAM have been listed in a recent review^1^; among them were the exact mechanism for resolution enhancement and a robust measurement of the achieved resolution. The enhancement of the numerical aperture was listed as the main contributing factor in resolution enhancement in MAM. Other factors included evanescent wave collection, photonic nanojet effects, resonant effects, and substrate and specimen-specific effects.

In an article published in this issue^28^, the investigation team performed numerical simulation using finite element method (FEM) to investigate the resolution enhancement mechanism in a microsphere-enhanced interferometry setup^29,30^. Although previous simulation works^31,32^ have been done, their model involves rigorous treatment of light scattering at the specimen’s surface and modeling the high NA objective lenses used for illumination and imaging. They considered full 3D conical Kӧhler illumination as well as conical imaging of the scattered waves by the MS. The resolution enhancement and magnification were studied with respect to the NA of the objective lens. Their case study was done for a 5-μm-diameter sphere with refractive index of 1.5 placed on a silicon sinusoidal phase grating with 25 nm peak-to-valley amplitude and 13.2 μm period. A 100× (0.9 NA) objective lens and monochromatic light at a non-resonant (440 nm) and resonant (480.76 nm) wavelength was considered; more simulation detail is provided in their previous work^33^. Their results confirmed that the enhancement of the NA is the main factor in resolution enhancement in MAM. No significant difference between the resonant and non-resonant wavelengths was observed.

The proposed method can be extended to conventional and confocal microscopies to help better understand and shed more light on MAM. Their approach can also be used to investigate parameters influencing MAM and find the optimum MS in terms of the refractive index, size, and index of the background medium^34^ for a given application.
